# Managing Early Childhood Caries: A Comparative Review of Preventive and Restorative Approaches

**DOI:** 10.7759/cureus.74704

**Published:** 2024-11-28

**Authors:** Afnan A Aljohani, Ahmad I Alarifi, Mubarak F Almoain, Faisal F Alrhaimi, Mashael T Alhejji, Nada W Gazzaz, Lulah S Ali, Hassan D Alammari, Rawan R Alwattban, Hanan M Alharbi, Bandar M Barnawi

**Affiliations:** 1 General Dentistry, Shaqra General Hospital, Riyadh, SAU; 2 General Dentistry, King Saud University, Riyadh, SAU; 3 General Dentistry, Aljouf University, Aljouf, SAU; 4 General Dentistry, Burydah Private Collage, Burydah, SAU; 5 General Dentistry, Imam Abdulrahman Bin Faisal University, Dammam, SAU; 6 General Dentistry, Armed Forces Hospital Southern Region, Khamis Mushait, SAU; 7 General Dentistry, Applied College, King Khalid University, Abha, SAU; 8 Dentistry, Applied College at Khamis Mushait, King Khalid University, Khamis Mushayt, SAU; 9 General Dentistry, Qassim University, Qassim, SAU; 10 Family Dentistry, Ministry of Health, Medinah, SAU

**Keywords:** early childhood caries, oral hygiene education, pediatric oral health, restorative techniques, silver diamine fluoride

## Abstract

Early childhood caries (ECC), one of the most common health problems among children aged six years and below, is considered present when one or more surfaces of a tooth are decayed, missing, or filled. Not only does ECC cause pain for a long time, but it also has short- and long-term health consequences in children. In order to treat ECC, holistic management that includes preventive, restorative, and prosthetic intervention is necessary.

This review, a key aspect of which is preventive strategy, stresses the need to lessen the rate of incidence of such conditions as ECC. In case preventive measures do not work, restorative care is considered. The paper discusses all of the available restoration options for paediatric patients. Furthermore, this review includes a detailed analysis of the different types of crowns used in the treatment of ECC, such as stainless-steel crowns (SSCs), resin-composite crowns, and zirconia crowns, and considers their advantages and disadvantages relevant to clinical practice.

Overall, this article calls for a well-rounded approach to treating the child’s dental cavity as an emerging progression of ECC. Through this interdisciplinary approach, dentists can significantly enhance oral health among children and help prevent the negative impact of caries on their quality of life.

## Introduction and background

Early childhood caries (ECC) is a global and multifactorial dental disease that has undesirable effects on the oral health of young children [1]. A diagnosis is made when a child, aged from birth to 71 months, shows evidence of decay in one or more primary teeth, which may include non-cavitated or cavitated lesions, teeth missing due to decay, or filled surfaces [2]. In addition to dental pain and discomfort, it has numerous complications, such as infections, difficulties in eating and speaking, and even systemic health issues. Psychosocially, it may result in a loss of self-esteem and quality of life for the child, as well as lasting implications for their school performance and social interactions [1].

The global prevalence of ECC is alarming, especially within non-privileged communities that have no access to dental services. The differences in socioeconomic status, education, and healthcare access increase the rate at which this disease is acquired by a person. Therefore, the path forward should include prevention and restorative measures to manage this condition as a public health problem in the community [1].

As it can be challenging to manage ECC, prevention is encouraged through methods such as educating the caregiver on proper oral hygiene techniques [3], consuming a well-balanced diet, and optimising fluoride exposure [4]. Regular visits to the dentist and prompt intervention can greatly minimise the likelihood of caries occurring in early childhood [5,6]. Restorations would be needed if these preventative strategies are insufficient to protect against ECC. Restoration techniques range from the minimum, which includes applying topical fluoride to the application of varnishes and sealants, to more intense interventions, such as fillings or crowning, depending on the severity level of the lesions. Additionally, if the tooth structure is destroyed at a higher level, prosthetic measures are used to help restore functionality and aesthetics [7].

Early childhood caries remains a prevalent health issue with significant physical, systemic, and psychosocial impacts, disproportionately affecting children from lower socioeconomic backgrounds. Despite advancements in dental care, significant disparities in ECC prevention and management persist, particularly in underserved regions. While numerous studies have explored specific aspects of ECC, a comprehensive review that synthesises both preventive and restorative approaches is essential for bridging the gap between current practices and unmet needs. This review aims to provide dental professionals with evidence-based strategies tailored to the unique challenges posed by ECC, contributing to improved oral health outcomes and quality of life for young children globally.

## Review

Methodology

A comprehensive literature search was conducted across PubMed, Scopus, Google Scholar, and reputable organizational websites to identify studies and resources addressing early childhood caries (ECC), including its prevalence, risk factors, preventive strategies, and restorative approaches. The search yielded a total of 140 resources: 55 articles from PubMed, 50 from Scopus, 30 from Google Scholar, and five documents from official websites, including the World Health Organization (WHO), the American Academy of Pediatric Dentistry (AAPD), and other pediatric dental associations. After removing 25 duplicates, the remaining 115 sources underwent further screening based on predefined inclusion and exclusion criteria.

Studies were included if they provided original data, systematic reviews, meta-analyses, or guidelines on ECC-related topics. Inclusion criteria focused on peer-reviewed articles, English-language publications, and resources published by recognized health organizations. Exclusion criteria omitted non-English studies, unpublished theses, conference abstracts, and sources not directly related to ECC. This process led to the exclusion of 45 sources, resulting in a total of 70 articles and documents being included in the final review.

To ensure comprehensiveness, the reference lists of included studies and relevant organizational reports were manually reviewed for additional resources. Cross-referencing citations from bibliographies and key documents further enhanced the scope of the review, ensuring the inclusion of the latest and most reliable information on ECC.

Understanding ECC

Characteristics of ECC

Early childhood caries is a common dental condition affecting young children, with a global prevalence estimated at 23.8% among those under 36 months and 57.3% among those aged 36 to 71 months [8]. Socioeconomic factors, dietary practices, and limited access to dental care are key determinants of its prevalence, particularly in underserved communities [1]. Early childhood caries typically begins with white spot lesions on the maxillary incisors, which result from enamel demineralization caused by bacterial acids. If untreated, these lesions progress to cavitated stages, leading to enamel breakdown, cavity formation, and symptoms such as pain and temperature sensitivity. Advanced cases may involve bacterial infiltration into deeper structures like dentin and pulp, often causing abscesses, swelling, and systemic complications [9,10]. Severe early childhood caries (S-ECC), which affects multiple teeth, can result in premature tooth loss, malocclusion, and developmental challenges [11,12].

The progression of ECC underscores its multifactorial nature, with microbial factors playing a central role. The primary pathogen associated with ECC is *Streptococcus mutans*, which thrives in dental plaque, particularly in the presence of fermentable carbohydrates. Other microorganisms, such as* Candida albicans*, may also contribute to disease progression [13]. Behavioral factors, including poor oral hygiene practices, high sugar consumption, and irregular dental visits, exacerbate the condition, highlighting the importance of preventive measures [10].

In addition to physical complications, ECC significantly impacts a child's quality of life. The condition interferes with essential activities like eating, speaking, and social interaction, often leading to reduced self-esteem and academic performance [1]. Furthermore, children from low-income families bear a disproportionate burden of ECC due to socioeconomic inequities, which limit access to dental preventive care and education [1]. Addressing ECC requires a comprehensive approach that includes early diagnosis, caregiver education, dietary modifications, and the use of fluoride to prevent disease progression and mitigate its long-term impacts.

Epidemiology of ECC

Prevalence and Risk Factors

Early childhood caries has been identified as a significant public health issue among children worldwide. The prevalence of ECC varies greatly across regions, depending on socioeconomic status, access to dental care, and dietary practices. A literature review indicates that in most developed countries, ECC prevalence ranges from 1% to 12% [14]. However, the prevalence can reach as high as 70% in less developed nations and among impoverished populations in wealthier countries. Regions with accessible preventive dental care, such as regular fluoride varnish applications and parental education programs, tend to report lower ECC prevalence [14,15]. In contrast, areas where preventive measures are less accessible often see higher rates of ECC, reflecting the effectiveness of prevention in managing this condition [3]. Furthermore, children from lower socioeconomic backgrounds are at a higher risk of developing ECC [15, 5].

In Europe, incidence rates vary, with approximately 11.4% of children affected in Sweden and between 7% and 19% in Italy [16, 17]. In the Middle East, ECC prevalence is particularly high, with rates of 76% reported in Palestine and 83% in the United Arab Emirates (UAE) [18, 19]. In Palestine, limited access to dental care, compounded by socioeconomic challenges, contributes significantly to these high rates [20]. Interestingly, despite being a wealthy nation, the UAE continues to report high ECC prevalence. This anomaly may be attributed to factors such as the increased consumption of sugary foods and beverages, inadequate oral hygiene practices among children, and insufficient utilization of preventive dental services, even when they are available [21]. In contrast, countries with robust preventive care programs, such as Sweden and the United States, report comparatively lower ECC rates, underscoring the importance of preventive strategies in managing ECC across diverse socioeconomic settings.

Surveys conducted in various countries indicate differing prevalence rates: 36% in Greece, 45.8% in Brazil, 51.9% in India, and 61.4% in Egypt. [22-25]. A systematic review concluded that ECC prevalence ranges from 2.1% in Sweden to 85.5% among children living in rural areas of China [26]. In the United States, the national prevalence of ECC is reported to range between 3% and 6%, aligning with rates observed in other Western countries [27, 28]. In certain Middle Eastern regions, where ECC prevalence is high, targeted preventive programs have been shown to reduce ECC incidence significantly, demonstrating the effectiveness of early intervention [1]. The age group most affected by ECC is three to four years, with research showing a significantly higher incidence among boys compared to girls between the ages of eight months and seven years [29].

Risk Factors for ECC

Several risk factors contribute to the development of ECC, including socioeconomic status, diet, and oral hygiene practices. Children from low socioeconomic backgrounds are particularly vulnerable to ECC due to limited access to dental care and lower parental education levels [5,6]. In some countries, preventive programs targeting low-income communities, such as subsidized dental care and education on oral hygiene, have shown the potential to reduce these disparities [30].

Diet plays a central role in ECC development, with high consumption of sugary beverages and snacks serving as a primary risk factor. Poor oral hygiene practices and infrequent dental visits further increase the likelihood of ECC. A cross-sectional survey conducted among school children in Saudi Arabia found that only 41.9% practiced good oral hygiene, underscoring the need for integrating dental health education into school curricula [5, 6].

Geographical Variations in ECC Prevalence

Variations in ECC prevalence across regions reflect disparities in access to dental care and the influence of cultural and dietary practices. Rural areas often report higher prevalence rates due to the limited availability of dental services, while urban regions may encounter challenges related to dietary habits and oral hygiene practices influenced by local traditions [6, 31, 32]. Addressing these differences is essential for designing equitable and targeted public health strategies to combat ECC effectively.

Etiology

The etiology of ECC is multifactorial, involving the interplay of bacteria, diet, host factors, and environmental influences. *Streptococcus mutans* is the primary bacterium responsible for ECC, producing acids that demineralize enamel, particularly in the presence of fermentable sugars [33, 34]. Frequent sugar consumption and nighttime feeding with sugary liquids significantly increase the risk of caries by promoting bacterial growth and acid production, particularly when combined with poor oral hygiene [35, 36]. Socioeconomic factors, such as limited access to dental care and maternal education, further influence ECC prevalence, especially in underserved communities [37]. Protective factors, such as adequate fluoride exposure and salivary function, play a crucial role in mitigating the risk of ECC [1, 4]. Additionally, genetic predispositions, including enamel defects or variations in saliva composition, may increase susceptibility to caries [38]. This multifaceted etiology underscores the importance of targeted preventive strategies, including caregiver education, dietary modifications, and fluoride programs, to combat ECC effectively.

Management of ECC

Managing ECC requires a dual approach, combining preventive measures to minimize the risk of caries development and restorative interventions to address existing lesions and complications. Preventive strategies, including caregiver education, dietary modifications, fluoride use, and sealants, aim to stop the onset or progression of caries. When prevention is insufficient, restorative approaches such as fillings, crowns, or extractions are employed to restore function and aesthetics while preventing further damage. This section delves into these management strategies, starting with preventive approaches, which form the cornerstone of ECC control.

Preventive Approaches

Preventive measures play a crucial role in the management of ECC and can significantly reduce its occurrence. These measures address risk factors at both the individual and community levels, emphasizing the need for adequate education, access to dental care, dietary changes, and the use of preventive agents [1,2,9].

Oral hygiene education: Educating caregivers about the importance of oral hygiene is essential for preventing ECC, as studies indicate that regular oral hygiene education can reduce caries incidence in young children by promoting healthier habits at home. Caregivers should be taught how to effectively brush children’s teeth using a soft-bristled toothbrush and fluoride toothpaste. It is important to begin brushing as soon as the first tooth erupts and to establish a consistent oral hygiene routine early in the child’s life [39].

In addition to tooth brushing, other oral hygiene methods can further enhance caries prevention. Flossing should be introduced once adjacent teeth make contact, as it removes plaque from interdental spaces where brushing cannot reach [1,2,9]. Tongue cleaning is another effective measure, as it helps reduce bacterial load and improve overall oral hygiene. For older children at high risk of caries, the use of alcohol-free fluoride mouth rinses can be considered, particularly in cases where dietary habits or medical conditions predispose them to enamel demineralization [9,12]. Caregivers should also encourage children to rinse with water after consuming sugary or acidic foods to minimize the risk of caries [1,2,9,12]. These additional methods complement tooth brushing and contribute to a comprehensive oral hygiene routine.

Behavioral changes: Behavioral changes are integral to the success of preventive strategies for managing ECC. Approaches like motivational interviewing have shown considerable promise in empowering caregivers to adopt healthier oral hygiene practices. Motivational interviewing involves collaborative conversations that encourage behavioral adjustments, fostering a sense of shared responsibility between healthcare providers and caregivers. This method has been demonstrated to improve compliance with preventive measures, such as routine brushing and dietary modifications, thereby reducing ECC incidence [39,5].

These behavioral approaches are particularly effective in underserved communities, where traditional educational programs may fall short due to cultural, logistical, or resource-based challenges. Motivational interviewing can bridge these gaps by tailoring interventions to the unique needs of caregivers, promoting sustainable oral health practices. For instance, caregivers exposed to motivational interviewing have reported increased adherence to recommended fluoride use and improved brushing frequency in their children, ultimately contributing to better oral health outcomes [12,5, 39].

Incorporating behavioral strategies within broader preventive efforts ensures a more holistic approach to ECC management, particularly in addressing barriers that may limit the success of conventional educational programs. Behavioral changes not only support the adoption of healthier practices but also reinforce their long-term sustainability, making them an essential component of ECC prevention [13,39].

Fluoride: Fluoride is one of the most effective agents for preventing dental caries, supported by extensive evidence. The AAPD recommends using a smear or grain of rice-sized amount of fluoride toothpaste for children under three years and a pea-sized amount for those over three years [40]. These guidelines aim to balance effective caries prevention while minimizing the risk of enamel fluorosis. In areas without fluoridated water, fluoride supplements, such as tablets or drops, may be prescribed based on individual risk assessments, typically at doses ranging from 0.25 mg to 1.0 mg per day depending on age and water fluoride levels [40]. Additionally, topical fluoride varnish is recommended two to four times per year for high-risk populations. Studies, including systematic reviews and meta-analyses, have demonstrated that fluoride varnish reduces caries incidence by approximately 43% in primary teeth and 37% in permanent teeth, emphasizing its significant role in high-risk populations [41, 42].

Sealants: Dental sealants are an effective measure for caries prevention, particularly on occlusal surfaces of molars, which are more prone to decay. Sealants made from glass ionomer cements (GICs) or resin-based materials are commonly used. Glass ionomer cement sealants are preferred when moisture control is challenging, as they release fluoride and provide additional protection [42]. Resin-based sealants, on the other hand, demonstrate higher retention rates and are suitable when adequate isolation can be achieved during application [42].

Studies indicate that sealants can reduce caries risk on primary molars by 76% at 24-48 months and up to 85% at 84 months [43]. The placement of sealants on primary molars is typically indicated for children at high risk of caries, particularly those with deep pits and fissures, enamel defects, or early signs of demineralization. Current evidence supports sealant placement shortly after the eruption of primary molars, as the occlusal surfaces are most vulnerable during this period [42]. For children, age and eruption status are critical factors in determining sealant application. Sealants are generally recommended for children between the ages of three and six years, as this is the period when primary molars are most at risk of developing caries [43].

Diet: Dietary habits play a significant role in the prevention of ECC. Specifically, a diet high in free sugars is a major risk factor for caries development. Evidence suggests that promoting nutritious foods and minimizing sugary snacks and drinks can reduce caries risk in young children. Healthy snacking behaviors, including the consumption of fruits and vegetables, can improve overall health and reduce the risk of caries [39]. Hydration is also important, and caregivers should encourage water as the primary beverage between meals instead of sugary drinks to minimize prolonged acid exposure to teeth [1,23,39].

Public health interventions targeting dietary habits have shown significant promise in reducing ECC prevalence. For example, limiting free sugar intake to below 10% of total daily caloric intake is recommended, as excess sugar consumption is closely linked to caries formation. Additionally, encouraging the inclusion of high-fiber fruits and vegetables in children's diets has been associated with improved salivary flow, which contributes to enamel demineralization [32].

Actionable strategies, such as educating parents and caregivers about the hidden sugars in processed foods and beverages, are critical. Governments and healthcare providers should collaborate on community-based programs that advocate for healthier diets to reduce ECC prevalence in underserved populations [35].

Brushing: Proper tooth brushing is one of the most effective measures for maintaining oral hygiene. The AAPD recommends that children brush their teeth twice daily with parental assistance until they develop adequate motor skills, typically around six years of age [39]. Supervision ensures that all tooth surfaces are thoroughly cleaned, reducing plaque buildup and preventing caries development [14]. Teaching caregivers the correct brushing technique is equally important. Caregivers should encourage gentle circular motions, which are effective in cleaning tooth surfaces without causing damage to the gums or enamel [11]. Starting proper brushing habits as soon as the first tooth erupts lays the foundation for long-term oral health [32]. These habits, combined with other preventive measures, play a critical role in maintaining children's oral hygiene and mitigating the risk of early childhood caries [2].

Toothpaste in caries prevention: Toothpaste is a cornerstone of dental caries prevention, combining mechanical plaque removal with chemical agents that support remineralization, reduce demineralization, and inhibit bacterial activity. Recent advancements in toothpaste formulations have introduced innovative therapeutic agents, expanding beyond fluoride to address specific oral health challenges.

Fluoride and advanced remineralization agents: Fluoride toothpaste remains the gold standard in caries prevention. Concentrations typically range from 1000 to 1500 ppm, effectively strengthening enamel through remineralization and reducing demineralization by forming fluorapatite, a more acid-resistant mineral than hydroxyapatite [40,44,45]. The American Dental Association recommends a smear-sized amount for children under three and a pea-sized amount for those aged three to six, balancing efficacy with the risk of fluorosis [44,46]. Studies have shown that fluoride toothpaste reduces caries prevalence by 24-30% across various populations [46,47]. While fluoride remains central to caries prevention, adjunctive technologies like casein phosphopeptide-amorphous calcium phosphate (CPP-ACP) and arginine-based formulations further enhance effectiveness, particularly in high-risk groups. Personalized toothpaste selection based on individual needs maximizes benefits and addresses specific oral health challenges [44,46,47]. Emerging remineralization technologies such as nano-hydroxyapatite (nHA) and calcium phosphate have gained attention for their ability to repair enamel. Nano-hydroxyapatite mimics the natural enamel structure, filling microscopic lesions and reinforcing tooth surfaces, while calcium phosphate provides essential ions for remineralization, particularly in high-risk groups [45,46].

Innovations in anticaries and antibacterial formulations: Recent toothpaste formulations incorporate novel technologies to address microbial activity and enhance remineralization. Arginine-based toothpastes increase salivary pH, creating an environment unfavorable for acidogenic bacteria while promoting enamel repair. Casein phosphopeptide-amorphous calcium phosphate acts as a reservoir of bioavailable calcium and phosphate ions for sustained remineralization, while xylitol disrupts bacterial metabolism, reducing plaque and acid production [44,45,47].

Toothpaste with antibacterial properties, such as those containing stannous fluoride or zinc, offer dual benefits: enamel protection and biofilm reduction. Stannous fluoride has demonstrated efficacy against caries and gingivitis due to its antibacterial properties, while zinc citrate and herbal extracts cater to the growing demand for natural alternatives [44-46]. These formulations are particularly valuable in managing high caries risk and improving gingival health in diverse populations.

Tailored toothpaste formulations for specific needs: Modern toothpaste formulations address a wide range of oral health needs, including hypersensitivity, enamel erosion, and aesthetics. Toothpaste containing potassium nitrate or strontium chloride alleviates dentinal hypersensitivity by blocking nerve responses, while low-abrasive formulations prevent further damage to the enamel in patients with erosion or restorations. Whitening agents, designed to remove surface stains, cater to aesthetic concerns without compromising enamel integrity [44-46]. Dental professionals play a pivotal role in guiding patients toward appropriate toothpaste formulations based on their unique risk profiles and oral health goals, ensuring comprehensive and effective care [44,46,47].

Probiotics: Probiotics have emerged as a promising adjunct in the prevention of ECC, targeting the microbial composition of the oral cavity. Specific strains such as *Lactobacillus rhamnosus* and *Bifidobacterium lactis* have demonstrated inhibitory effects on cariogenic bacteria, particularly *Streptococcus mutans* [48-50]. Clinical studies have explored various probiotic delivery systems, including chewing tablets, lozenges, milk, and yogurt, to enhance their accessibility and efficacy [48,49,51]. These probiotic interventions aim to modulate the oral microbiome by reducing pathogenic bacterial load and promoting a balanced microbial environment conducive to oral health.

Furthermore, probiotics have been associated with anti-inflammatory effects, offering additional benefits beyond microbial modulation. Research highlights their role in reducing oral inflammation, enhancing mucosal immunity, and potentially lowering the incidence of caries in children at high risk [48,49]. For example, a study found that probiotic yogurt consumed regularly over six months significantly reduced S. mutans counts in a randomized trial [48, 51]. Another study demonstrated that probiotics like *Lactobacillus paracasei* SD1 improved oral immunity by enhancing human neutrophil peptide production, contributing to reduced bacterial colonization and caries risk [48,49]. A recent study by Hedayati-Hajikand et al. [52] demonstrated the efficacy of ProBiora3® probiotics in reducing caries increment among preschool children. When used as an adjunct to fluoride toothpaste, the intervention resulted in a significant 75% reduction in early enamel demineralization compared to a placebo group over a year-long period. This finding reinforces the potential role of probiotics in augmenting traditional preventive measures.

While probiotics show promise, their long-term efficacy in ECC prevention requires further investigation, particularly in establishing standardized formulations, effective dosages, and delivery mechanisms. Research must also explore their application across diverse populations to ensure reliability and scalability in caries management. However, their inclusion as part of a comprehensive oral health strategy underscores the potential of probiotics to improve outcomes in high-risk pediatric populations [48-53].

Silver diamine fluoride (SDF): Silver diamine fluoride has emerged as a potent agent for managing ECC through its ability to both arrest existing caries and prevent new lesions. The active components, silver ions, and fluoride, work synergistically to combat bacterial activity and reinforce tooth structure. Silver ions exhibit strong antimicrobial properties by inhibiting bacterial growth and disrupting biofilm formation, while fluoride promotes enamel remineralization, thereby fortifying the tooth against further demineralization [41, 54].

The application of 38% SDF has shown high efficacy in halting the progression of carious lesions, particularly in young children and populations with limited access to dental care. Randomized controlled trials have demonstrated that biannual applications of SDF effectively arrest caries in up to 80% of cases over a two-year period, outperforming other topical fluoride agents in high-risk populations [54]. This evidence underscores its potential as a reliable and cost-effective intervention for reducing the burden of ECC.

Silver diamine fluoride's ease of application and noninvasive nature make it particularly suitable for young children, including those who are uncooperative or have special needs. However, one notable drawback is the black staining of arrested carious lesions, which may pose esthetic concerns, particularly for anterior teeth. Despite this limitation, caregivers often prioritize its benefits over esthetics, especially when SDF is the only viable option in resource-constrained settings [41, 54].

While SDF is primarily used for caries arrest, emerging evidence suggests its potential role in preventing new carious lesions. Preliminary studies indicate that the antimicrobial and fluoride-releasing properties of SDF may create an oral environment less conducive to caries development. Nevertheless, further longitudinal studies are required to establish standardized protocols for its preventive application [54].

By addressing the dual needs of caries prevention and arrest, SDF offers a versatile and practical solution for managing ECC. Its integration into preventive care strategies, particularly in underserved populations, represents a significant step toward reducing the global burden of childhood dental disease [41, 54].

Impact of screen time and digital media: The pervasive presence of digital media in children’s lives has raised significant concerns about its impact on oral hygiene and overall health. Excessive screen time, particularly when problematic or unstructured, has been linked to multiple adverse oral health outcomes, including a higher prevalence of plaque, gingival inflammation, and dental caries [55-57]. Studies show that children with prolonged screen exposure are more likely to snack mindlessly on sugary and acidic foods, which promotes bacterial growth and plaque formation, thereby increasing the risk of cavities [58]. Additionally, the reduced physical activity associated with high screen time can lower saliva flow, essential for clearing food particles and bacteria, which further exacerbates dental risks [51]. Notably, problematic screen exposure (PSE) has been specifically associated with higher scores in plaque and gingival indices, reflecting poorer oral hygiene among preschool children with frequent media use [57].

Screen use can also disrupt regular oral hygiene routines, as children engrossed in digital devices may be less likely to brush and floss consistently, leading to plaque buildup and an increased risk of dental decay [56,59]. Moreover, the sedentary behaviors encouraged by prolonged screen time may indirectly contribute to oral health issues, as low physical activity levels impair natural protective functions such as saliva production [51]. Furthermore, behaviors such as teeth grinding (bruxism) and jaw clenching, sometimes observed during intense screen activities like gaming, can lead to wear on teeth, jaw pain, and even temporomandibular joint (TMJ) disorders [58].

Guidelines from the World Health Organization and the AAPD recommend limiting screen time, especially for children under five, to mitigate these risks. Parents play a critical role in reducing the impact of screen time on oral health by setting screen limits, encouraging regular brushing and flossing, and providing healthier snack options during screen use [57,59]. When managed thoughtfully, structured and educational screen content can also help reinforce positive oral hygiene habits, aligning with preventive health strategies [58].

Restorative approaches to ECC

While prevention is the cornerstone of ECC management, situations may arise where preventive measures are insufficient. In such cases, restorative treatment becomes essential to manage caries, preserve dental structure, and ensure functionality. Prevention strategies, including regular dental check-ups [5,6], fluoride applications [4], and dietary counseling [3], play a vital role in minimizing the risk of caries. However, when preventive measures fail, restorative techniques such as fillings, crowns, and extractions are employed to address the consequences of caries and maintain the integrity of the child’s dental structure [7]. This section reviews restorative approaches, highlighting their indications, advantages, and limitations to provide practitioners with evidence-based options for holistic patient care.

Traditional Restorative Techniques

Traditional restorative techniques are fundamental to managing caries in primary teeth. These methods typically involve the removal of decayed tissue and the restoration of the tooth's structure to preserve function and aesthetics while preventing further damage. Examples of such techniques include the use of composite resins, GIC, and resin-modified glass ionomer cements (RMGICs) [60]. These materials offer a range of benefits, from esthetic restoration to fluoride release, and are chosen based on factors such as the extent of decay, the child’s age and cooperation level, and the tooth’s location. These approaches provide reliable solutions tailored to the specific needs of pediatric patients.

Composite Resins

Composite resins are widely used in pediatric dentistry due to their excellent aesthetic properties and versatility. Comprising a resin matrix and inorganic fillers, they are capable of closely matching the color of natural teeth, making them ideal for anterior restorations. These materials bond effectively to both enamel and dentin, offering durability and utility in primary teeth. However, their performance is influenced by several factors, including their clinical effectiveness, durability, and cost considerations [60-62].

Studies demonstrate a survival rate of approximately 80.6% for composite restorations in primary teeth over an 18-month period, with better outcomes in anterior teeth due to reduced occlusal stress ​[62]. While composites offer superior aesthetics and wear resistance compared to other materials, they are highly technique-sensitive. Proper moisture control during application is critical, as insufficient isolation can compromise the bond strength and long-term success of the restoration​ [62].

In terms of cost, composite resins are generally more expensive than materials such as GIC, which might limit their use in certain socioeconomic contexts. Furthermore, while they do not release fluoride-like GIC or RMGIC, their aesthetic and structural advantages make them a preferred choice for anterior restorations in primary teeth. Clinical trials have shown that composites can effectively restore functionality and aesthetics, but the technical requirements for their successful application remain a significant limitation​ [62].

Glass Ionomer Cements

Glass ionomer cements are widely recognized for their ability to release fluoride, promote enamel demineralization, and provide antibacterial effects, which are particularly beneficial for children at high risk of caries. These materials chemically bond to both enamel and dentin, making them highly effective in environments where moisture control is challenging, such as in young or uncooperative pediatric patients. Their versatility and biocompatibility also make them a preferred choice for atraumatic restorative treatment (ART), especially in underserved or rural settings with limited access to dental resources [62, 63].

Clinical studies have demonstrated the durability and effectiveness of GICs, reporting survival rates of 93% for single-surface ART restorations and 62% for multi-surface restorations over two years. These findings highlight their suitability for minimally invasive techniques aimed at preserving dental structures while reducing secondary caries risks through continuous fluoride release [62]. Additionally, GICs are less technique-sensitive than resin-based materials, further enhancing their applicability in resource-constrained scenarios or when working with less cooperative patients [41, 53].

Despite these strengths, GICs have limitations, including lower compressive strength and wear resistance compared to resin-based alternatives, which restrict their use in high-load-bearing restorations, such as the occlusal surfaces of molars. However, advancements in high-viscosity GIC formulations have significantly improved their mechanical properties, making them more resilient for broader applications [41, 62]. These enhanced formulations continue to address key gaps, particularly in cases where durability and fluoride release are critical considerations.

In comparison to composite resins, GICs provide the dual advantages of fluoride release and tolerance to wet environments, though they may lack the aesthetic qualities and strength required for anterior restorations. Such properties position GICs as an indispensable tool in managing caries in primary teeth, particularly for high-risk populations or in settings where access to advanced dental care is limited [63].

Resin-Modified Glass Ionomer Cements

Advancements in restorative materials have led to the development of RMGICs, which address some of the limitations of traditional GICs. By incorporating resin into their composition, RMGICs combine the fluoride-releasing properties and chemical adhesion of GICs with enhanced physical properties, including better fracture resistance, wear tolerance, and aesthetic appeal [64, 65].

One of the most significant features of RMGICs is their dual-curing mechanism, which utilizes both chemical and light-curing processes. This provides increased flexibility during application and ensures optimal curing even in challenging clinical situations. Their enhanced durability makes RMGICs particularly suitable for primary molars with moderate caries, where restorative materials are subjected to significant occlusal stress [62, 65].

Studies have demonstrated the clinical efficacy of RMGICs, with survival rates of approximately 89% for single-surface restorations and 75% for multi-surface restorations in primary molars over two years. These results underscore their reliability for caries management in children, particularly in settings with limited access to regular dental follow-up [57, 58]. Moreover, their ability to bond chemically to enamel and dentin ensures effective sealing, reducing the risk of microleakage and secondary caries [56].

Fluoride release is another critical advantage of RMGICs. This feature not only aids in remineralization but also provides ongoing protection against caries, making these materials especially beneficial for high-risk pediatric populations. Compared to composite resins, RMGICs are more forgiving in environments with less stringent moisture control, broadening their applicability in real-world clinical settings [62, 63, 65].

However, despite these strengths, RMGICs are not without limitations. Their mechanical properties, while improved over traditional GICs, remain inferior to composite resins, restricting their use in high-stress areas. Additionally, the need for light-curing equipment can pose challenges in resource-limited settings, though their overall ease of use and caries-preventive benefits often outweigh these drawbacks [64,65].

By offering a balance between functionality and practicality, RMGICs serve as a versatile option for restoring primary teeth affected by moderate caries. Their combination of fluoride release, chemical bonding, and improved physical properties makes them an invaluable asset in pediatric restorative dentistry, particularly for managing caries in underserved populations [41, 62-65].

Other Restorative Techniques

Silver-modified atraumatic restorative treatment (SMART) restorations: This combines the principles of minimally invasive caries management with restorative techniques tailored for underserved populations. This approach is particularly effective for managing dental caries in areas with limited access to conventional dental care [62,63]. The SMART technique utilizes the manual removal of decayed tissue followed by the application of fluoride-releasing restorative materials, such as GIC. These materials provide dual benefits: chemical bonding to the tooth structure and the continuous release of fluoride, which aids in remineralization and protects against secondary caries [62,63]. Studies have demonstrated the effectiveness of SMART restorations in primary teeth, showcasing their potential to achieve functional and durable outcomes in high-risk pediatric populations. The technique’s non-invasive nature and cost-effectiveness make it an ideal choice for community-based dental programs targeting young children. While aesthetic concerns associated with discoloration in some variations of this approach may arise, the clinical advantages of SMART restorations outweigh these drawbacks in most cases [41,63-67].

Table 1 presents a comparison of restorative materials for the management of ECC.

**Table 1:** Comparison of restorative materials for ECC management GIC: glass ionomer cement; ECC: early childhood caries

Material	Aesthetic quality	Fluoride release	Moisture tolerance	Durability	Ideal use cases	Limitations
Composite resins	High [61]	None [61]	Low [61]	Moderate [61]	Anterior teeth [61]	Requires moisture control [61]
Glass ionomer cement	Moderate [62]	Yes [41]	High [63]	Moderate [62]	High-risk cases, young patients [62]	Less aesthetic [62]
Resin-modified GICs	Moderate [64]	Yes [63]	High [63]	High [64]	Molars with moderate caries [65]	Requires light-curing [64]

Crowns in ECC management

When preventive measures and conventional restorative methods fall short in managing extensive carious lesions, crowns serve as a reliable option for restoring both function and aesthetics in primary teeth. Crowns offer comprehensive protection by covering the entire tooth, thereby preventing fractures, maintaining dental arch integrity, and supporting adjacent teeth. They are particularly valuable in cases of severe decay, trauma, or after pulp therapy, where the tooth's structure and vitality require reinforcement and long-term durability [68, 69].

Indications for Crown Placement

Crown placement is recommended in various clinical scenarios to address the unique challenges presented by extensive decay and compromised dental structures. For teeth with large carious lesions that cannot be effectively restored with traditional fillings, crowns provide a durable and reliable solution [66, 69, 70]. Additionally, they offer essential protection for patients at high risk of future caries, reducing the need for repeated interventions [66,68]. Structurally compromised teeth, whether due to trauma, caries, or developmental defects such as enamel hypoplasia, benefit significantly from the reinforcement provided by crowns [64, 65]. In cases where pulp therapy, such as pulpotomy or pulpectomy, has been performed, crowns restore both the structural integrity and functional capacity of the affected tooth [69,71,72]. Crowns are also valuable for managing non-carious lesions, mitigating the effects of severe bruxism, or addressing developmental anomalies [71]. Furthermore, crowns can act as abutments for space maintainers, preserving alignment and spacing in the dental arch until the eruption of permanent teeth [70]. These versatile applications underline the critical role of crowns in maintaining oral health and functionality in pediatric patients.

Types of crowns

Several types of crowns are available for ECC management, including stainless steel crowns (SSCs), esthetic crowns, zirconia crowns, and prefabricated crowns. Each type has its own distinct properties, advantages, and limitations.

Stainless Steel Crowns

Stainless steel crowns are among the most reliable restorative options for managing extensive caries in primary teeth, known for their exceptional durability, cost-effectiveness, and ability to preserve tooth structure. Commonly used for primary molars, SSCs are especially indicated in cases of multi-surface caries, teeth requiring pulpotomy or pulpectomy, and children with a high risk of caries. These crowns are also effective in managing developmental defects such as enamel hypoplasia or amelogenesis imperfecta, providing long-term protection until natural exfoliation [69, 70].

Clinical studies demonstrate the superiority of SSCs over other restorative materials, with survival rates exceeding 90% over extended follow-up periods [71]. Stainless steel crowns are particularly suited for high-stress applications due to their resilience against occlusal forces and their minimal tooth preparation requirements, which preserve sound tooth structure and simplify the procedure for young or anxious patients. Despite their many advantages, the metallic appearance of SSCs can limit their use in anterior teeth. However, modifications such as the open-faced SSC technique have been developed to improve aesthetics while retaining the benefits of these crowns [70].

Esthetic Crowns

Esthetic crowns, such as composite resin and zirconia crowns, are preferred for anterior restorations in pediatric dentistry due to their ability to closely mimic the natural appearance of teeth. Composite resin crowns are highly customizable, allowing practitioners to match the color of adjacent teeth, making them ideal for cases where esthetics are prioritized. However, their durability may be limited in areas with high occlusal stress, leading to fractures or discoloration over time. Despite this, studies report high parental satisfaction with the aesthetic outcomes of composite crowns, particularly for restoring anterior teeth [69, 71].

Zirconia crowns offer enhanced durability and aesthetics, making them suitable for both anterior and posterior teeth. These crowns are biocompatible and highly resistant to plaque accumulation, ensuring long-term functionality and aesthetics. However, they require more extensive tooth reduction compared to composite crowns and come at a higher cost, which may limit their accessibility in some cases [67, 71]. The choice between composite and zirconia crowns should be guided by the specific clinical and aesthetic needs of the patient, as well as considerations such as cost and longevity. Esthetic crowns play a crucial role in pediatric restorative dentistry by addressing both functional and psychological needs, particularly in cases of severe anterior tooth damage or decay [69, 71].

Zirconia Crowns

Zirconia crowns are a premier option in pediatric dentistry, offering an ideal combination of exceptional strength and aesthetics. These ceramic crowns closely mimic the appearance of natural teeth, providing superior resistance to wear and fracture, making them suitable for both anterior and posterior restorations. They are particularly beneficial in cases requiring post-endodontic treatment or for children where both durability and aesthetics are critical factors [72]. Compared to SSCs, zirconia crowns offer enhanced biocompatibility and aesthetic outcomes, with reduced plaque accumulation and minimal gingival irritation. These features make them an excellent choice for anterior teeth restorations or families prioritizing the visual appeal of dental restorations. However, zirconia crowns require a more technique-sensitive placement process, including precise tooth preparation and the use of adhesive cement to achieve optimal retention and marginal adaptation [68, 72]. The cost of zirconia crowns, significantly higher than SSCs or composite resin crowns, can limit their accessibility in some settings. Their placement often demands greater clinical expertise and specialized equipment, making them less feasible in resource-limited environments. In such cases, SSCs remain a practical and cost-effective alternative, particularly for posterior restorations where aesthetics are less critical. Composite resin crowns may also be a suitable option for anterior teeth in patients with financial constraints, offering acceptable aesthetics at a lower cost [68, 72]. While zirconia crowns represent an unparalleled option for combining aesthetics and durability, their use should be guided by careful case selection. Considerations such as the patient’s age, caries risk, tooth location, and the financial feasibility of treatment are essential for optimizing outcomes. For families with the resources and preference for high-quality restorations, zirconia crowns provide long-lasting solutions that meet both functional and aesthetic demands [67, 72].

Prefabricated Crowns

Prefabricated crowns are a cornerstone of pediatric dentistry, offering ready-made solutions for restoring primary teeth. Available in materials such as stainless steel, composite resin, and zirconia, they cater to various clinical and aesthetic needs [68, 69].

Stainless steel crowns: Ideal for posterior teeth due to their durability and cost-effectiveness. These crowns provide reliable protection for extensively decayed teeth or those treated with pulp therapy but may not appeal aesthetically for anterior restorations [69, 70].

Composite resin crowns: Preferred for anterior restorations due to their customizable aesthetic qualities. While less durable than zirconia crowns, they are a cost-effective and visually appealing option for less stress-bearing areas [68, 70].

Zirconia crowns: Offer a balance of strength and aesthetics, making them suitable for both anterior and posterior teeth. These crowns are biocompatible and resistant to wear, though they require precise placement and are relatively expensive [71, 72].

Prefabricated crowns require minimal preparation and reduced chair time, making them a practical option for young patients. Their material versatility allows practitioners to tailor treatments to the specific needs of the child, ensuring optimal outcomes in both function and appearance [69, 72].

Table 2 compares crown types for ECC management by durability, aesthetics, indications, cost, and limitations.

**Table 2:** Comparison of crown types for ECC management ECC: early childhood caries

Crown type	Durability	Aesthetic quality	Indications	Cost-effectiveness	Limitations
Stainless steel crowns	High [69]	Low [70]	High-risk molars [69]	High [70]	Poor aesthetics for anterior teeth [70]
Esthetic crowns	Moderate [67]	High [68]	Anterior teeth [68]	Moderate [67]	Less durable in molars [68]
Zirconia crowns	High [72]	High [72]	Both anterior and posterior teeth [72]	High [72]	Higher cost [72]
Prefabricated crowns	Moderate-high [69]	Variable [69]	Posterior or anterior teeth, based on material [68]	High [69]	Limited customization [69]

Discussion

Management of ECC must adopt a holistic approach that integrates both preventive and restorative measures. Early intervention is essential, as it allows dentists to positively influence the trajectory of a child’s oral health. A comprehensive management strategy, which includes caregiver education, routine access to dental care, and the application of preventive measures, has the ability to shift the balance in favor of controlling the challenges posed by ECC [41,45,61].

Preventive strategies are the cornerstone of ECC management. Educating caregivers about oral hygiene practices is crucial. Caregivers must understand the importance of brushing children's teeth with fluoride toothpaste twice daily, starting from the eruption of the first tooth [44,46]. Families also benefit from education about dietary modifications that can lower the risk of ECC [32,39]. This includes avoiding sugary treats and beverages [35], providing healthy, nutritious foods, encouraging water as the primary beverage, and adhering to good eating habits.

Public health initiatives play a pivotal role in enhancing accessibility to preventive care for underserved populations. Programs such as mobile dental clinics, school-based fluoride varnish applications, and caregiver education campaigns address barriers like cost and geographic access [41,47,61]. By equipping families with the knowledge and tools to prioritize oral health, these initiatives reduce reliance on restorative measures and promote sustainable caries prevention [41,47,61]. Collaboration with local organizations, especially schools, ensures that these efforts reach families where they are most likely to engage.

Routine dental visits are another essential component of ECC prevention. These visits allow for the early detection and resolution of caries and other dental issues. Dentists can provide tailored advice based on each child’s specific needs and circumstances during these appointments. Effective preventive measures, such as the application of GIC sealants on primary molars [42] and SDF [47], are especially beneficial for high-risk populations. These interventions not only protect the teeth from caries but also foster lifelong good oral health practices.

Restorative approaches, while essential for addressing established caries, are more resource-intensive and invasive than preventive measures. Techniques such as ART, which minimize discomfort and avoid the need for local anesthesia, are particularly beneficial for young or anxious children [62,63]. Additionally, crowns, including stainless steel and esthetic options, offer both functional and aesthetic solutions tailored to pediatric needs [62,64,65]. However, the long-term success of these interventions is highly dependent on caregiver compliance with follow-up visits and oral hygiene practices [45,61]. Treatment decisions must be individualized, taking into account the child’s age, level of cooperation, extent of caries, and the clinical situation.

Managing ECC effectively requires a collaborative approach that includes dental practitioners, caregivers, and community resources working together to promote sound oral health habits [73,74]. Dentists play a pivotal role not only in treating children but also in guiding caregivers on how to maintain oral health. Community engagement through workshops, outreach programs, and school-based activities enhances awareness and equips families with the necessary knowledge to make informed decisions about their children’s dental care. Access to fluoride treatments, dental screenings, and educational materials through community resources is critical for filling gaps in care, especially for underserved populations. Advocacy efforts are essential to ensure that policy changes promote equitable access to oral healthcare for all children [75,76].

The integration of technology offers additional potential for improving oral health education and management. For instance, mobile applications can be used to remind caregivers of dental hygiene routines, track dietary habits, and facilitate access to telehealth services. Social media platforms can also play a significant role in disseminating information and fostering discussions about oral health within communities [75]. Long-term parental involvement remains crucial for sustainable change in ECC management [77]. Parents must be active participants in their children’s oral health journey and foster healthy habits that will last a lifetime. Modeling good oral health practices within the family reinforces these behaviors and promotes a health-focused family culture beyond simple hygiene.

The future of ECC management lies in further research into its etiology and the development of effective prevention and treatment strategies. Innovations, such as exploring the role of probiotics in oral health, require more investigation. Continuous education and training of dental practitioners will ensure that they stay informed about the latest procedures and products in pediatric dentistry, thereby improving patient care. Effective policy reform is also crucial, including more funding for preventive dental services in schools, integrating dental care into comprehensive healthcare for children, and addressing social determinants of health that contribute to disparities in oral health outcomes. All children should have the opportunity to enjoy healthy smiles, making it crucial to foster a cultural shift toward preventive care and early intervention for long-term oral health promotion [76].

## Conclusions

Early childhood caries remains a major public health concern, requiring both preventive and restorative approaches. Preventive measures such as caregiver education, dietary changes, fluoride applications, and emerging interventions like probiotics and silver diamine fluoride are vital in reducing caries risk. Restorative options, including composite resins, GICs, and crowns, provide effective solutions, with innovations like RMGICs and zirconia crowns enhancing durability and aesthetics.

To address ECC globally, funding preventive care programs and integrating dental education into public health initiatives are crucial. Expanding access to cost-effective, minimally invasive techniques like SMART restorations and prioritizing community-based interventions can significantly reduce ECC prevalence and improve long-term outcomes.
